# Molecular Characteristics of Extended-Spectrum β-Lactamases in Clinical Isolates from *Escherichia coli* at a Japanese Tertiary Hospital

**DOI:** 10.1371/journal.pone.0064359

**Published:** 2013-05-15

**Authors:** Hisakazu Yano, Mina Uemura, Shiro Endo, Hajime Kanamori, Shinya Inomata, Risako Kakuta, Sadahiro Ichimura, Miho Ogawa, Masahiro Shimojima, Noriomi Ishibashi, Tetsuji Aoyagi, Masumitsu Hatta, Yoshiaki Gu, Mitsuhiro Yamada, Koichi Tokuda, Hiroyuki Kunishima, Miho Kitagawa, Yoichi Hirakata, Mitsuo Kaku

**Affiliations:** 1 Department of Infection Control and Laboratory Diagnostics, Internal Medicine, Tohoku University Graduate School of Medicine, Sendai, Miyagi, Japan; 2 Department of Otolaryngology, Head and Neck Surgery, Tohoku University Graduate School of Medicine, Sendai, Miyagi, Japan; 3 Department of Bacteriology, BML Inc., Kawagoe, Saitama, Japan; 4 Department of Regional Cooperation for Infectious Diseases, Tohoku University Graduate School of Medicine, Sendai, Miyagi, Japan; St. Petersburg Pasteur Institute, Russian Federation

## Abstract

The prevalence of ESBL has been increasing worldwide. In this study, we investigated the molecular characteristics of ESBL among clinical isolates of *Escherichia coli* from a Japanese tertiary hospital. A total of 71 consecutive and nonduplicate clinical isolates of ESBL-positive *E. coli* collected at Tohoku University Hospital between January 2008 and March 2011 were studied. The antimicrobial susceptibility profile of these strains was determined. PCR and sequencing were performed to identify genes for β-lactamase (*bla*_TEM_, *bla*_SHV_, *bla*_OXA-1-like_, and *bla*_CTX-M_) and plasmid-mediated quinolone resistance determinants (PMQR). The isolates were also analyzed by pulsed-field gel electrophoresis (PFGE) and multilocus sequence typing (MLST). Of the 71 strains, 68 were positive for CTX-M, 28 were positive for TEM, four were positive for OXA-1, and one was positive for SHV. Sequencing revealed that CTX-M-14 was the most prevalent (31/71), followed by CTX-M-27 (21/71) and then CTX-M-15 (9/71). Of the 28 TEM-positive strains, one was TEM-10 and the rest were TEM-1. One SHV-positive strain was SHV-12. The 21 CTX-M-27-producing isolates were divided into 14 unique PFGE types, while the 9 CTX-M-15 producers were divided into 8 types. Based on MLST, 9 CTX-M-14 procedures, 19 CTX-M-27 procedures, and 8 CTX-M-15 producers belonged to ST131. Thirty-five (94.6%) of the 37 ST131 *E. coli* strains showed resistance to levofloxacin, which was a higher rate than among non-ST131 strains (63.6%). Among ESBL-producing isolates, one, two, and six possessed *qnrB, qnrS, qepA*, and *aac(6′)-Ib-cr*, respectively. Of the 6 isolates with *aac(6′)-Ib-cr*, 4 carried the CTX-M-15 gene. Our data suggest that CTX-M-15-producing *E. coli* ST131 has emerged as a worldwide pandemic clone, while CTX-M-27 (a variant of CTX-M-14) is also spreading among *E. coli* ST131 in Japan.

## Introduction

The ability of bacteria to produce extended-spectrum β-lactamases (ESBL) that hydrolyze penicillins, cephalosporins, and monobactams has resulted in intractable infections and serious consequences for infection control. Recently, the prevalence of ESBL procedures has been increasing and infections caused by these bacteria have become an emerging public health concern worldwide [1], [2].

ESBL can be classified into three main types, which are designated as TEM, SHV, and CTX-M. The CTX-M type of ESBL can be further classified into three groups, which are CTX-M-1, CTX-M-2, and CTX-M-9. In the 1990s, ESBL were generally found in *Klebsiella pneumonia* (TEM or SHV types) and most isolates were from nosocomial infections. Since 2000, however, the worldwide distribution of ESBL producers has shifted towards *Escherichia coli* with CTX-M type and isolates are obtained from both inpatients and outpatients [3]–[7]. In particular, CTX-M-15 (which belongs to the CTX-M-1 group) is widely distributed around the world [2]. In Japan, *E. coli* producing CTX-M type ESBL have also been emerging. In the early 2000s, the dominant CTX-M group underwent a shift from CTX-M-2 to CTX-M-9 [8].

There have also been reports about quinolone resistance among ESBL producers [7]. Quinolone resistance is usually caused by chromosomal mutations, but can also be related to plasmid-mediated quinolone resistance (PMQR) genes, including *qnrA*, *qnrB*, *qnrC*, *qnrS*, *qepA,* and *aac(6′)-Ib-cr* [9]. Several studies have indicated that the emergence of PMQR determinants in ESBL-producing *Enterobacteriaceae* poses a global threat [10], [11]. However, there have been few Japanese reports about the detection of PMQR and the prevalence of PMQR determinants among ESBL producers in Japan remains unclear [12].

In this study, we investigated the molecular characteristics and epidemiology of clinical isolates of ESBL-producing *E. coli* obtained at a Japanese tertiary hospital. This work was presented in part at the 52th Interscience Conference on Antimicrobial Agents and Chemotherapy (ICAAC), San Francisco, 2010.

## Materials and Methods

### Bacterial Strains

A total of 71 (2.9%) consecutive and non-duplicate clinical isolates of ESBL-producing *E. coli* were collected from among 2,488 *E. coli* isolates at Tohoku University Hospital during the period from January 2008 to March 2011. Each isolate was identified by using the VITEK 2 system (Sysmex bioMérieux Co., Ltd., Tokyo, Japan), and initial screening for ESBL was done with the VITEK 2 Advanced Expert System (Sysmex bioMérieux Co.) according to the manufacturer’s instructions. ESBL production was confirmed by the combined disk test according to CLSI guidelines [13], [14]. Among the 71 ESBL-producing strains of *E. coli*, 37 (52.1%) were isolated from urine, nine (12.7%) from sputum, five (7.0%) from blood, 3 (4.2%) from stools and abscesses, two (2.8%) from the pharynx and a wound, and 10 (14.1%) from other sites.

### Antimicrobial Susceptibility Testing

The minimum inhibitory concentration (MIC) of various antimicrobial agents was determined by the agar dilution method according to CLSI guidelines [13], [14]. The following antimicrobial agents were tested in this study: ampicillin, piperacillin, piperacillin-tazobactam, cefotaxime, ceftazidime, cefepime, cefoxitin, imipenem, meropenem, aztreonam, levofloxacin, gentamicin, and amikacin. Quality control for the MIC analyses was performed with *E. coli* ATCC 35218 and *E. coli* ATCC 25922.

### Detection of ESBL Genes and DNA Sequencing

PCR was performed with TaKaRa *Ex Taq* (Takara Bio Inc., Otsu, Japan) to identify ESBL genes, including *bla*_TEM_, *bla*_SHV_, *bla*_OXA-1-like_, and *bla*_CTX-M_ [15], [16]. For CTX-M-positive strains, the CTX-M group was determined by PCR using CTX-M-1, CTX-M-2, and CTX-M-9 group-specific primers [16]. PCR products of the TEM, SHV, OXA-1-like, CTX-M-1 group, CTX-M-2 group, and CTX-M-9 group genes were sequenced on both strands with an ABI3730XL analyzer (Applied Biosystems, Foster City, CA, USA). BLAST version 2.2.24 (http://blast.ddbj.nig.ac.jp/) was used to process the sequencing data and identify genes. To differentiate *bla*_CTX-M-15_ from *bla*_CTX-M-28_ in the CTX-M-15-positive strains, the published primer pair was used for PCR and sequencing [17].

### PMQR Gene Detection and DNA Sequencing

Detection of *qnrA*, *qnrB*, *qnrC*, *qnrS*, *aac(6′)-Ib*, and *qepA* in the isolates was performed by PCR, as described previously [15]. The *aac(6′)-Ib* amplicons were also sequenced to identify the -cr variant, as described above.

### Pulsed-Field Gel Electrophoresis

For CTX-M-15- and CTX-M-27-positive strains, evaluation of chromosomal polymorphism was done by pulsed-field gel electrophoresis (PFGE) using the *Xba*I restriction enzyme (Takara Bio Inc.), as described previously [18]. Electrophoresis was performed on 1% PFGE agarose gel with a CHEF-DR III system (Bio-Rad Laboratories, Richmond, CA, USA), and electrophoretic patterns were analysed with GelCompar II software (Applied Maths, Kortrijik, Belgium). Isolates that showed >85% similarity were considered to reside within a single cluster [18].

### Multilocus Sequence Typing

For CTX-M-14-, CTX-M-15-, and CTX-M-27-positive strains, multilocus sequence typing (MLST) was performed using seven housekeeping genes (*adk, fumC, icd, purA, gyrB, recA,* and *mdh*) according to the method of Jørgensen et al. [19]. DNA sequence variations were analyzed by using a MLST database for *E. coli* (http://mlst.ucc.ie/mlst/dbs/Ecoli).

### Statistical Analysis

Statistical significance was evaluated by the chi-square test with Yates’ correction or Fisher’s exact test, and a p value of less than 0.05 was considered to be significant.

### Ethics

This study focused on bacterial strains that were isolated for treatment. In addition, this study was completely anonymous and no identifiable information was obtained. According to the ethical guidelines for epidemiological studies released by the Ministry of Health, Labour, and Welfare in Japan [20], ethical approval and written or verbal informed consent are not required for this type of study.

## Results

### Antimicrobial Susceptibility Profile

The results of antimicrobial susceptibility testing are shown in Table 1. Meropenem was the most active agent (100% susceptibility; MIC range: ≤0.03–0.5 mg/L). Over 90% of the 71 strains showed intermediate resistance or resistance to ampicillin, piperacillin, cefotaxime, and aztreonam. In addition, 62.0% and 67.6% of the isolates showed intermediate resistance or resistance to ceftazidime and cefepime, respectively, while 32.4% displayed intermediate resistance or resistance to piperacillin-tazobactam.

**Table 1:** Susceptibility profile of ESBL-positive *E. coli.*

Antimicrobial Agent	MIC (µg/ml) for all isolates (n = 71)			Percent non-susceptible a)
	min	max	MIC90	
ampicillin	256	256	256	100
piperacillin	64	256	256	100
piperacillin-tazobactam	2/4–	256/4	256/4	32.4
cefotaxime	8	256	256	100
ceftazidime	1	256	64	93.0
cefepime	2	256	64	67.6
cefoxitin	4	256	64	62.0
imipenem	0.03	1	0.25	0
meropenem	0.03	0.5	0.03	0
aztreonam	2	256	128	90.1
levofloxacin	0.13	256	128	78.9
gentamicin	1	256	256	35.2
amikacin	4	256	16	9.9

Furthermore, 78.9% of all isolates demonstrated intermediate resistance or resistance to levofloxacin, while intermediate resistance or resistance to gentamicin and amikacin was noted in 35.2% and 9.9%, respectively.

### ESBL Typing and Antimicrobial Susceptibility Profile

Among the 71 strains, 68 strains (95.8%) were positive for CTX-M, 28 strains (39.4%) were positive for TEM, four strains (5.6%) were positive for OXA-1-like, and one strain (1.4%) was positive for SHV. Among the 68 CTX-M-positive strains, 13 were from the CTX-M-1 group, one was from the CTX-M-2 group, and 54 were from the CTX-M-9 group.

DNA sequencing revealed that CTX-M-14 was most common (31 strains: 45.6%), followed by CTX-M-27 (21 strains: 30.9%) and then CTX-M-15 (9 strains: 13.2%). The only CTX-M-2 group-positive strain was CTX-M-2. Of the 28 TEM-positive strains, one was TEM-10 and the other 27 were TEM-1, and all 27 TEM-1-positive isolates possessed the CTX-M gene. One SHV-positive strain was SHV-12, and this isolate possessed CTX-M-14. All of the four OXA-1-like were OXA-1, and these isolates possessed CTX-M-15.

All 9 of the CTX-M-15-positive isolates showed intermediate resistance or resistance to ceftazidime, as did 18 (85.7%) of the 21 CTX-M-27-positive isolates. These rates of resistance were higher than those found in the other CTX-M-positive isolates (50%). The antimicrobial susceptibility profiles of CTX-M-15-positive strains are shown in Table 2.

**Table 2:** β-lactamases, PMQR, and antimicrobial susceptibility profiles of CTX-M-15-positive isolates.

No. of strains	β-lacrtamases	PMQR	ST	PIPC	PIPC/TAZ	CAZ	CTX	CFPM	AZT	MEPM	LVFX	GM	AMK
9	CTX-M-15, TEM-1	–	ST131	?256	128	32	?256	32	64	?0.03	2	128	8
24	CTX-M-15, OXA-1	*aac(6′)-Ib-cr*	ST131	?256	16	64	?256	32	64	?0.03	32	64	32
40	CTX-M-15, OXA-1	*aac(6′)-Ib-cr*	ST131	?256	8	32	?256	16	64	?0.03	32	128	16
45	CTX-M-15, TEM-1, OXA-1	*aac(6′)-Ib-cr*	ST131	?256	128	8	64	16	64	?0.03	64	4	32
55	CTX-M-15, OXA-1	*aac(6′)-Ib-cr*	ST131	?256	128	128	?256	128	128	?0.03	32	4	32
56	CTX-M-15	–	ST131	?256	?256	?256	?256	?256	?256	?0.03	32	4	32
57	CTX-M-15	–	ST131	?256	?256	?256	?256	?256	?256	?0.03	64	?256	16
62	CTX-M-15	–	ST131	?256	?256	?256	?256	?256	?256	?0.03	64	2	16
72	CTX-M-15, TEM-1	–	ST58	?256	64	64	?256	128	128	?0.03	0.13	1	4

### PMQR Gene Typing

One isolate was positive for *qnrB,* one for *qnrS,* and two for *qepA* in this study. None of the isolates were positive for *qnrA* or *qepA.* Among a total of 71 isolates, 7 carried *aac(6′)-Ib* genes, and sequencing revealed *aac(6′)-Ib-cr* in six of these 7 strains.

### Analysis of PMQR-Positive Strains

Among the 10 PMQR-positive isolates, six possessed resistance genes from the CTX-M-1 group. Of the six isolates with *aac(6′)-Ib-cr* genes, four (66.7%) carried the CTX-M-15 gene. The SHV-12-positive strains possessed *qnrB* genes. All of the PMQR-positive strains were resistant to levofloxacin (MIC: 32 - ≥ 256 mg/L).

### PFGE

**Figure 1:**
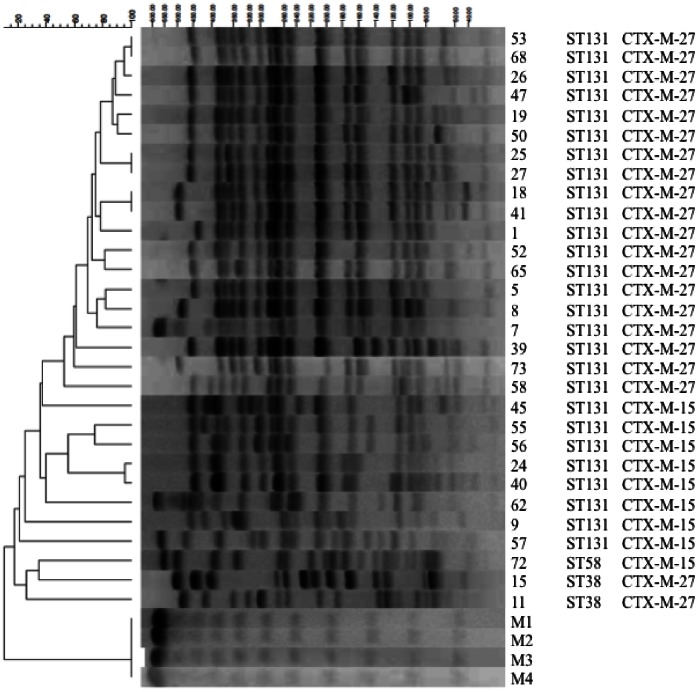
MLST and PFGE of *Xba*I-digested genomic DNA from CTX-M-15- and CTX-M-27-producing *E. coli* strains. The 9 CTX-M-15-producing isolates were divided into 8 unique PFGE types. The 21 CTX-M-27-producing isolates were divided into 14 unique PFGE types, with the most common type being found in 6 patients. M1∼4: Lambda Ladder.

Figures 1 shows a dendrogram and PFGE of *Xba*I-digested genomic DNA from 9 CTX-M-15-producing and 21 CTX-M-27-producing strains of *E. coli*. The 9 CTX-M-15-producing isolates were divided into 8 unique PFGE types, and the commonest type was found in 2 patients. The 21 CTX-M-27-producing isolates could be divided into 14 unique PFGE types, with the most common type being found in 6 patients.

### MLST

Based on the results of MLST analysis, 8 (88.9%) of the 9 CTX-M-15-producing *E. coli* strains belonged to ST131 and one strain (11.1%) belonged to ST58. Among the 21 CTX-M-27-producing strains, 19 (90.5%) belonged to ST131 and two (9.5%) belonged to ST38. The CTX-M-14-producing strains were divided into 8 unique MLST types, and 10 (32.2%) of these strains were ST131 (Table 3). Of the 37 ST131 isolates, 35 (94.6%) showed resistance to levofloxacin, and this rate of resistance was significantly higher than that among non-ST131 strains (58.3%).

**Table 3:** MLST types of CTX-M-14-, CTX-M-15-, and CTX-M-27-positive strains.

CTX type	n	Sequence type								
		10	38	58	68	92	95	131	357	648
CTX-M-14	31	1	6		5	1	2	10	2	4
CTX-M-15	9			1				8		
CTX-M-27	21		2					19		

## Discussion

Currently, the CTX-M type is predominant among ESBL producers around the world. Many epidemiological studies of CTX-M type ESBL have been performed in different countries [1], [2], and CTX-M-15-producing *E. coli* ST131 have spread worldwide [2]. In the present study, a total of nine CTX-M-15-positive strains (eight belonging to ST131) were isolated. There has been one previous report about detection of CTX-M-15- producing *E. coli* ST131 in Japanese patients [21]. The present study demonstrated that these strains can be frequently isolated in a Japanese tertiary hospital.

In the present study, four of the six *aac(6′)-Ib-cr* positive strains possessed CTX-M-15, and we demonstrated a significant association between the CTX-M-15 and *aac(6′)-Ib-cr* genes. It has been reported that isolates with the *aac(6′)-Ib-cr* gene often possess a CTX-M-15-producing plasmid [5], [22]. This suggests that spread of the *aac(6′)-Ib-cr* gene might occur concurrently with the CTX-M-15 gene. The *aac(6′)-Ib-cr* gene also confers resistance to aminoglycosides, so CTX-M-15 producers can develop multidrug resistance. In addition, most *E. coli* ST131 isolates identified around the world show resistance to quinolones confirmed by chromosomal mutations. In our study, 35 (94.6%) of the 37 *E. coli* ST131 isolates showed resistance to levofloxacin, and 7 of the 8 CTX-M-15-producing *E. coli* ST131 strains were resistant to levofloxacin. Quinolones are frequently used to treat infections among outpatients in many countries, including Japan, but there is concern that these drugs will not be effective against CTX-M-15 producers and will apply selection pressure to these isolates.

The 9 CTX-M-15-producing isolates could be divided into 8 unique PFGE types, while the 21 CTX-M-27-producing isolates were divided into 14 unique PFGE types with the most common type being found in 6 patients. These results suggested that certain clones were spreading in our hospital and that nosocomial infection was occurring, so improved infection control and surveillance is required.

We found 21 isolates that produced CTX-M-27 in this study. CTX-M-27 is a variant of CTX-M-14, which only differs by the substitution D240G [23]. Based on MLST analysis, CTX-M-14-producing strains were divided into 8 unique MLST types, and 10 (32.2%) of these strains were ST131. Most of the CTX-M-27-producing strains (19 strains: 90.5%) also belonged to ST131 (Table 3). Furthermore, the 14 unique clones of CTX-M-27-positive strains identified by PFGE analysis, 12 belonged to ST131. We previously reported that CTX-M-27 producers were frequently isolated in the clinical setting in Japan [21]. There have been no reports about a cluster of CTX-M-27-producing pathogens in other countries, so CTX-M-27-producing *E. coli* may have arisen in Japan due to a point mutation of the CTX-M-14 gene in *E. coli* ST131.

Several case reports of unusually severe or fatal extraintestinal infections due to *E. coli* ST131 [24]-[27] have suggested that the rapid and extensive emergence of such strains may be partly due to high virulence compared with other *E. coli* types. In addition, it was previously demonstrated that CTX-M-27 confers stronger resistance to CAZ than CTX-M-14 [23]. Thus, we have to be concerned that CTX-M-27-producing *E. coli* ST131 could become predominant over CTX-M-14 in Japan because of its high virulence and selection pressure due to use of CAZ.

A limitation of this study is that we did not investigate the CTX-M-8 and CTX-M-25 groups among CTX-M-type ESBL groups. However, these two groups have not yet been identified in Japan. The dominant group of ESBL-producing *E. coli* was CTX-M-2 until 2000, while CTX-M-9 has been the dominant group since 2000. This study showed increasing emergence of CTX-M-15 from the CTX-M-1 group, which was rarely reported previously in Japan. The dominant type of ESBL may change again in the future, so that investigation of the CTX-M-8 and CTX-M-25 groups, as well as rarely identified PER, VEB, and IBC type ESBL, may be necessary.

In conclusion, this study revealed that CTX-M-15-producing *E. coli* ST131 (a worldwide pandemic clone) has emerged in Japan. Our findings also suggest that CTX-M-27 (a variant of CTX-M-14) is spreading among clinical isolates of *E. coli* ST131 in the Japanese tertiary hospital setting.
